# An Atypical Presentation of Acute Pulmonary Embolism With Severe Acute Respiratory Syndrome Coronavirus 2 (SARS-CoV-2) Pneumonia

**DOI:** 10.7759/cureus.8249

**Published:** 2020-05-23

**Authors:** Nasha Elavia, Nishant Sharma, Si Li, Yichen Wang, Bojana Milekic

**Affiliations:** 1 Internal Medicine, The Wright Center for Graduate Medical Education, Scranton, USA; 2 Center for Cellular Engineering, National Institutes of Health, Bethesda, USA

**Keywords:** coronavirus, sars-cov-2, pulmonary embolism, thromboembolism, covid-19

## Abstract

Clinical presentation and severity of the severe acute respiratory syndrome coronavirus 2 (SARS-CoV-2) varies greatly amongst patients, as supported by recent literature. This poses an ongoing challenge in the diagnostic and therapeutic approach for managing these patients. Here, we would like to describe a case of acute bilateral pulmonary embolism (PE) presenting with atypical gastrointestinal symptoms in a patient with SARS-CoV-2 infection. This atypical presentation of PE is unique to our case and highlights the significance of a high index of clinical suspicion for SARS-CoV-2 and its associated thrombogenic effect, even in patients with atypical symptoms.

## Introduction

Although knowledge about the effects of the novel severe acute respiratory syndrome coronavirus 2 (SARS-CoV-2) has been emerging, so far, the most commonly documented reason for hospitalization was respiratory distress [1]. Proinflammatory states, such as acute infections, were known to be associated with an increased risk of thromboembolic events. Venous thromboembolism is an uncharacteristic complication of the SARS-CoV-2, and only few descriptions of potential correlation exist in the literature [2]. Newly discovered atypical features of the SARS-CoV-2 infection continue to be frequently reported, while increasing clinician’s awareness and mindfulness for uncommon presentations. Here, we would like to describe a case of acute bilateral pulmonary embolism (PE) in a patient with SARS-CoV-2 pneumonia who mainly presented with gastrointestinal symptoms.

## Case presentation

**Figure 1:**
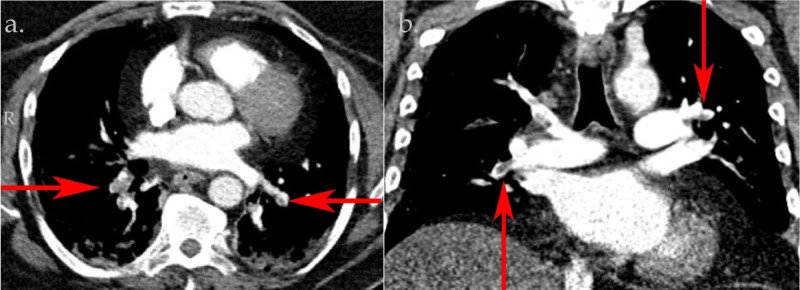
Pulmonary computed tomography angiography obtained on the day of hospital admission. Axial image (a) and coronal image (b) demonstrating bilateral filling defects of the pulmonary artery (red arrows).

A 64-year-old male with a past medical history notable for diabetes mellitus type 2, obstructive sleep apnea treated with nocturnal continuous positive airway pressure ventilation (CPAP) and glaucoma presented to the emergency department with diarrhea and loss of appetite. The patient reported having four to five episodes of watery bowel movements per day, which started about seven days prior to admission. He denied any nausea, vomiting, abdominal pain, blood in stool, fever or any respiratory complaints of chest pain, shortness of breath or cough. Social history was negative for smoking, recent travel, sick contact exposure, immobilization, hospitalization or recent antibiotic use. On presentation, he was tachypneic at 20 breaths per minute and hypoxic with an oxygen saturation of 81% while breathing ambient air. His physical exam revealed bilateral basilar rhonchi, but otherwise he did not appear in any acute respiratory distress. Laboratory studies were notable for lymphopenia (absolute lymphocyte count 0.85 K/uL) along with significantly elevated lactate dehydrogenase (624 U/L), ferritin (2,237 ng/mL) and C-reactive protein (66 mg/L) levels. Chest X-ray revealed bilateral airspace opacification as seen with SARS-CoV-2. A nasopharyngeal swab for SARS-CoV-2 was positive. The pretest probability of PE calculated using the Wells score was less than 4, which was unlikely for PE. Bilateral lower extremity duplex was negative for deep venous thrombosis (DVT). D-dimer level was 1.52 ng/mL FEU. In the light of significant discrepancy between severe hypoxia and the absence of respiratory symptoms or a respiratory viral syndrome, a pulmonary CT angiogram (CTA) was performed, which confirmed acute bilateral PE extending from the distal right main pulmonary artery into all right lobes along with patchy ground-glass opacities consistent with SARS-CoV-2 pneumonia (Figure 1).

The patient was admitted to a negative pressure room and started on anticoagulation with heparin. He was eventually discharged home on an oral novel anticoagulant and continuous oxygen at 4 liters/minute via nasal cannula.

## Discussion

The SARS-CoV-2 pandemic is challenging and, in many cases, an overwhelming situation for the medical community. Research on the characteristics of this novel coronavirus is evolving every day as cases of SARS-CoV-2 continue to emerge with a multitude of clinical presentations. So far, fever is the most commonly reported symptom (approximately 88% of cases), followed by cough (68%), vomiting (5%) and diarrhea (3.8%) [3]. Up to 15% of patients develop complications of severe interstitial pneumonia leading to acute respiratory distress syndrome (ARDS), multiorgan failure, disseminated intravascular coagulation and death [3]. 

SARS-CoV-2 belongs to the family of genus Betacoronavirus. It shares similar imaging and clinical features with severe acute respiratory syndrome (SARS) and Middle East respiratory syndrome coronavirus (MERS). Chen et al. recently published findings of the thrombogenic effects amongst SARS-CoV-2 infected patients compatible with the literature from Singapore and the SARS epidemic from 2004 [4,5]. In contrast to our case, patients with suspected PE have been reported to have overlapping symptoms of SARS-CoV-2 [5]. Our patient however presented mainly with gastrointestinal symptoms, which have been reported with SARS-CoV-2; however, with significant hypoxia in the absence of a respiratory viral syndrome although with a low pretest probability for PE, we decided to further evaluate the patient for hypoxia. The presence of gastrointestinal symptoms and cardiopulmonary complications are reported in patients with SARS-Cov-2; however, the presence of gastrointestinal symptoms in SARS-CoV-2 complicated with PE in the absence of an overlapping respiratory viral syndrome makes our case unique. Risk assessment scores and laboratory data are widely used to assist in clinical decision making; nevertheless, the importance of clinicians' degree of suspicion and mindfulness for atypical presentations and complications cannot be overstated, especially in a currently evolving disease like SARS-CoV-2 infection.

At many centers across the United States, inflammatory marker levels are now routinely checked in SARS-CoV-2 patients. Studies have shown that elevated D-dimer levels correlate with the severity of SARS-CoV-2 illness, poor prognosis, higher likelihood of admission to the intensive care unit (ICU) and even death [3,6]. Although our patient did not have symptoms of a viral respiratory syndrome, an elevated D-dimer level prompted us to do a chest CTA. The decision was supported by the literature (algorithm by Zuckier et al. published in April 2020 suggests obtaining a chest CTA in SARS-CoV-2 patients with elevated D-dimer levels if there are no contraindications) [7]. Additionally, patients with severe SARS-CoV-2 disease, including those requiring invasive mechanical ventilation and intensive care, are known to have an increased risk of venous thrombosis [8]. Whether this thrombogenic phenomena is due to the virus itself or the acute inflammation in critically ill patients is not clear. As such, algorithms have been developed but the decision for therapeutic anticoagulation in SARS-CoV-2 patients at this time remains largely individualized, accounting for patient and physician factors. Further studies are required to aid in developing evidence-based guidelines for therapeutic anticoagulation in patients with SARS-CoV-2 depending on patient characteristics and severity of the infection.

## Conclusions

Cases of SARS-CoV-2 continue to emerge with atypical presentations such as the case highlighted here. Therefore, it is important for all physicians (and particularly, the emergency department) to have a high clinical suspicion for both SARS-CoV-2 and venous thrombosis and include these in the differential diagnoses and early identification of the disease.

## References

[1] Danzi GB, Loffi M, Galeazzi G, Gherbesi E (2020). Acute pulmonary embolism and COVID-19 pneumonia: a random association?. Eur Heart J.

[2] Xie Y, Wang X, Yang P, Zhang S (2020). COVID-19 complicated by acute pulmonary embolism. Radiol Cardiothorac Imaging.

[3] Guan WJ, Ni ZY, Hu Y (2020). Clinical characteristics of coronavirus disease 2019 in China. N Engl J Med.

[4] Chen N, Zhou M, Dong X (2020). Epidemiological and clinical characteristics of 99 cases of 2019 novel coronavirus pneumonia in Wuhan, China: a descriptive study. Lancet.

[5] Chong PY, Chui P, Ling AE (2004). Analysis of deaths during the severe acute respiratory syndrome (SARS) epidemic in Singapore: challenges in determining a SARS diagnosis. Arch Pathol Lab Med.

[6] Tang N, Bai H, Chen X, Gong J, Li D, Sun Z (2020). Anticoagulant treatment is associated with decreased mortality in severe coronavirus disease 2019 patients with coagulopathy. J Thromb Haemost.

[7] Zuckier LS, Moadel RM, Haramati LB, Freeman LM (2020). Diagnostic evaluation of pulmonary embolism during the COVID-19 pandemic. J Nucl Med.

[8] Ullah W, Saeed R, Sarwar U, Patel R, Fischman DL (2020). COVID-19 complicated by acute pulmonary embolism and right-sided heart failure [Epub ahead of print]. JACC Case Rep.

